# Comparison of Different Mandibular Jawlines Classifications on Transoral Endoscopic Thyroidectomy for Papillary Thyroid Carcinoma: Experiences of 690 Cases

**DOI:** 10.3389/fendo.2022.842148

**Published:** 2022-02-17

**Authors:** Xing Yu, Yuancong Jiang, Yujun Li, Qionghua He, Lei Pan, Peifeng Zhu, Yong Wang, Ping Wang

**Affiliations:** ^1^ Department of Thyroid Surgery, The Second Affiliated Hospital, Zhejiang University School of Medicine, Hangzhou, China; ^2^ College of Medicine, Zhejiang University, Hangzhou, China

**Keywords:** mandibular jawlines, length of jay, transoral endoscopic thyroidectomy, papillary thyroid carcinoma, surgical outcomes

## Abstract

**Background:**

The influences of patients’ different mandibular jawlines on transoral endoscopic thyroidectomy *via* vestibular approach (TOETVA) have not been described before. The objective of this study was to introduce a new classification to assess different mandibular jawlines, and to evaluate the effects on TOETVA in terms of safety, feasibility, and postoperative feelings in the treatment of papillary thyroid carcinoma (PTC).

**Methods:**

The crossing angle of esthetic plane and mandibular plane was defined as Wang Angle, used to assess patients’ different mandibular jawlines. Mandibular classifications of A (angle: 80° ~ 110°), B (angle > 110°), and C (angle < 80°) types were compared to evaluate the surgical outcomes of TOETVA by a retrospective study. 690 patients of PTC who received TOETVA were included in this study, which were divided into three groups according to mandibular classifications.

**Results:**

Clinicopathological characteristics of the patients including age, gender, body mass index, tumor size, Hashimoto thyroiditis were similar in the three groups. Patients’ length of jay in group C was significantly longer than group A and group B (*P* < 0.01). The ratios of using suspension system in group C were significantly higher than group A and group B (*P* < 0.01). The scores of postoperative visual analogue scale (VAS) and ratios of mandibular swell in group C were significantly higher than group A and group B (*P* < 0.01). There was no significant difference in the three groups regarding surgical outcomes, including postoperative vocal cord paralysis, hypocalcemia, serum white blood cells and C-reactive protein levels.

**Conclusions:**

The Wang angle and mandibular jawline classifications were firstly introduced in TOETVA. All the patients of class A, B, and C mandibular jawline can achieve safe and effective surgical outcomes in the treatment of PTC with TOETVA. Patients of class C need more assistance of suspension system, would experience higher scores of VAS, and higher ratios of mandibular swell compared with class A and B.

## Introduction

Transoral endoscopic thyroidectomy is new that does not cause neck scarring and requires a smaller subcutaneous flap elevation than that of remote access thyroid surgery methods (1). Because of these advantages above, there has been a rapid development of transoral endoscopic thyroidectomy *via* vestibular approach (TOETVA) in the past 10 years (2). However, the influences of patients’ different mandibular jawlines on TOETVA have not been described before (3). The angle’s classifications of malocclusion were initially used to assess the mesio-distal relationships of the dental arches (4), which was modified by us and used to assess the mandibular jawlines. The objective of this study was to introduce a new classification of mandibular jawlines into transoral endoscopic thyroidectomy, and to evaluate the effects on TOETVA in terms of safety, feasibility, surgical outcomes, and postoperative feelings in the treatment of papillary thyroid carcinoma (PTC).

## Materials and Methods

### Patients’ Enrollment

Between January 2015 and June 2020, we retrospectively enrolled 690 patients with PTC who underwent total thyroidectomy or ipsilateral thyroidectomy and lymph node dissection of central compartment in the Second Affiliated Hospital, Zhejiang University School of Medicine. All enrolled patients had cosmetic requirements, and they chose the operation of TOETVA. Patients were divided into three groups according to the different types of mandibular jawlines. The clinicopathological characteristics such as age, gender, body mass index (BMI), tumor size, multiple lesions ratio, and Hashimoto’s thyroiditis ratio were compared in the three groups. This study was approved by the ethical committee of the Second Affiliated Hospital of Zhejiang University School of Medicine.

### Introduction of Wang Angle and Mandibular Jawlines Classifications

**Figure 1:**
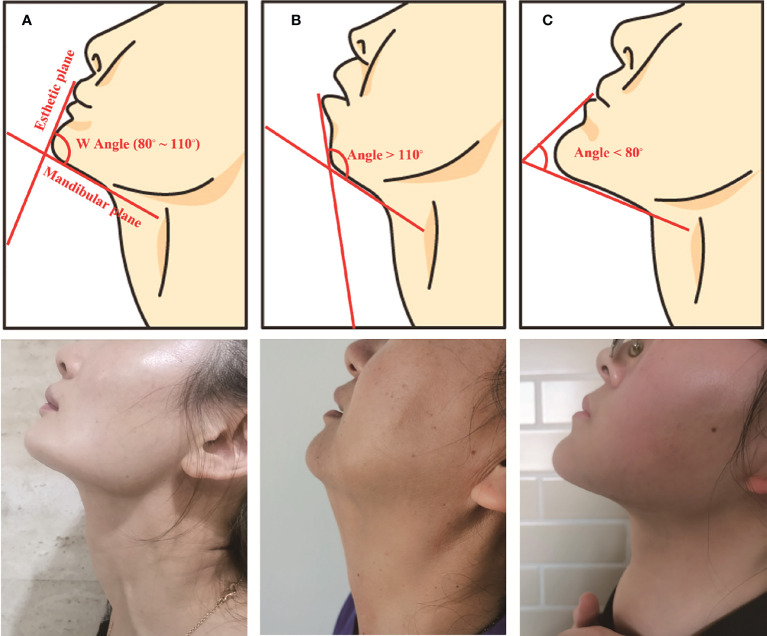
Schematic diagram and realistic photos of the three classifications: **(A)** (angle: 80° ~ 110°), **(B)** (angle > 110°), and **(C)** (angle < 80°) types.

The crossing angle of esthetic plane and mandibular plane was defined as Wang Angle **(** **Figure 1** **)**. Patients’ different mandibular jawlines were classified into three types according to the degree of Wang Angle, including A (angle: 80° ~ 110°), B (angle > 110°), and C (angle < 80°) types. This new classification was firstly introduced in TOETVA. As shown in the **Figure 1**, patients’ different mandibular jawlines were exhibited with schematic diagrams and realistic photos. The Wang Angle of each patient was defined as the crossing of esthetic plane and mandibular plane (red line), which was used to assess the different mandibular jawline classifications.

### Measure the Length of Jaw

**Figure 2:**
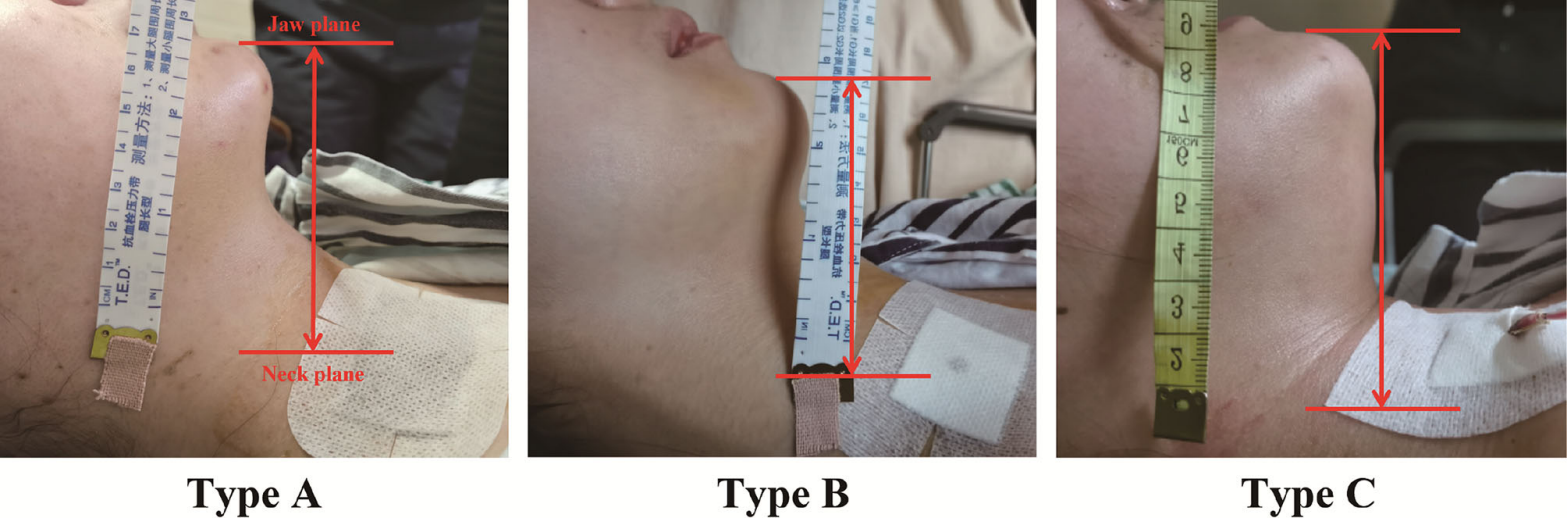
The length of jay was measured from anterior neck plane to the anterior border plane of jaw (red line) in the three groups.

The length of jaw was measured in the A, B, and C types of mandibular jawlines. The length of jaw is a quantitative index, which may be useful to evaluate the level of difficulty to maintain the surgical space in TOETVA. As shown in **Figure 2**, patients were chosen horizontal position, and the length of jaw was defined as the distance from anterior neck plane to the anterior border plane of jaw (red line). The length was measured by a flexible ruler, which was analyzed and compared in the three groups.

### Procedures of TOETVA

The procedures of TOETVA were briefly described in the following. A 10-mm incision for the camera port was cautiously made in the middle of the vestibule and frenulum. Another two 5-mm trocars were applied through the mucosa incision at the level of the first premolars for auxiliary use. A 30° angled camera was then advanced through the 10-mm port. Ultrasonic coagulation devices (Harmonic Scalpel, Ethicon Endosurgery, USA) were used to manage thyroid surrounding vessels and the inferior thyroid arteries (5). Thyroid gland was completely resected, and central node dissection (CND) was conducted including the prelaryngeal, pretracheal, and paratracheal areas (6).

### Comparison of Surgical Outcomes

Preoperative laryngoscope, thyroid hormones, parathyroid hormone (PTH) and calcium were recorded 3 days before surgery. Postoperative laryngoscope, laboratory tests including PTH, calcium, white blood cell (WBC), and C-reactive protein (CRP) were examined at the day next to the operation. Thyroid hormones, PTH, and calcium were detected at one month postoperatively. Patients receive postoperative review every 3 months after surgery, thyroid function and cervical B ultrasound were included each time.

### Statistical Analysis

The results are presented as number (%) and average ± SD appropriate. Data were analyzed by one-way ANOVA, Welch ANOVA, student *t*-test, the *χ2* test, Fisher’s exact test, and non-parametric Wilcoxon-Mann-Whitney test appropriately using SPSS 20.0 software (SPSS Inc., Chicago, IL, USA). A *p* value less than 0.05 was considered to be statistically significant.

## Results

### Clinicopathologic Characteristics

The clinicopathologic characteristics were summarized in **Table 1**. This study enrolled 690 patients, comprising 342 (Type A), 281 (Type B), and 67 (Type C) patients respectively. Patient’s age, sex ratio, and BMI were similar in the groups of type A, B, and C. Tumor characteristics were compared in the three groups, and there was no significant difference in the max tumor size, multiple lesions ratio, and Hashimoto’s thyroiditis ratio. Total thyroidectomy ratio was similar between the three groups of type A, B, and C respectively. The length of jaw was compared and has significant difference in the three groups (*P* < 0.01). In the pairwise comparison, the length of jaw in group C was significantly more than group A (7.26 ± 0.23 *vs*. 6.85 ± 0.23, *P* < 0.01), and group B (7.26 ± 0.23 *vs*. 6.17 ± 0.42, *P* < 0.01). And the length of jaw in group A was also more than group B significantly (6.85 ± 0.23 *vs*. 6.17 ± 0.42, *P* < 0.01).

**Table 1:** Comparation of clinicopathological characteristics in the groups of type A, B, and C.

	Type A (n = 342)	Type B (n = 281)	Type C (n = 67)	*P value*
Age (years)	37.3 ± 9.8	37.6 ± 9.9	35.8 ± 9.2	0.423
Male (%)	90 (26.3%)	74 (26.3%)	25 (37.3%)	0.159
BMI (kg/m^2^)	23.7 ± 3.7	23.7 ± 3.7	23.9 ± 3.8	0.825
Max tumor size (cm)	1.23 ± 0.59	1.20 ± 0.61	1.31 ± 0.71	0.175
Multiple lesions (%)	81 (23.7%)	88 (31.3%)	22 (32.8%)	0.065
Hashimoto’s thyroiditis (%)	100 (29.2%)	97 (34.5%)	25 (27.3%)	0.238
Total thyroidectomy (%)	111 (32.5%)	98 (34.9%)	25 (37.3%)	0.675
Bilateral CND (%)	108 (31.6%)	96 (34.2%)	19 (28.4%)	0.606
Length of jaw (cm)	6.85 ± 0.23	6.17 ± 0.42	7.26 ± 0.23	<0.01

BMI, body mass index; CND, central node dissection.

### Postoperative Feelings

The ratio of mandibular swell and visual analogue scale (VAS) feeling were compared in the three groups. As shown in **Table 2**, the ratio of mandibular swell was compared and has significant difference in the three groups (*P* < 0.01). In the pairwise comparison, the ratio of mandibular swell in group C was significantly more than group A (10.4% *vs*. 1.2%, *P* < 0.01), and group B (10.4% *vs*. 0.7%, *P* < 0.01). While there was no significant difference in the comparison of mandibular swell between group A and group B (1.2% *vs*. 0.7%, *P* = 0.560). The scores of VAS were compared and has significant difference in the three groups (*P* < 0.01). In the pairwise comparison, the scores of VAS in group C was significantly more than group A (2.1 ± 0.6 *vs*. 1.7 ± 0.5, *P* < 0.01), and group B (2.1 ± 0.6 *vs*. 1.7 ± 0.5, *P* < 0.01). While there was no significant difference in the comparison of VAS scores between group A and group B (1.7 ± 0.5 *vs*. 1.7 ± 0.5, *P* = 0.980).

**Table 2:** Effective assessment of surgical results in the groups of type A, B, and C malocclusion.

	Type A	Type B	Type C	*P value*
	(n = 342)	(n = 281)	(n = 67)	
EMG changes (%)	28 (8.2%)	21 (7.5%)	2 (3.0%)	0.330
Transient vocal cord paralysis (%)	7 (2.0%)	9 (3.2%)	1 (1.5%)	0.563
Postoperative PTH (pg/ml)	34.7 ± 16.5	36.0 ± 18.4	35.7 ± 18.9	0.642
Postoperative calcium	2.09 ± 0.14	2.11 ± 0.13	2.08 ± 0.13	0.360
numbness in limbs (%)	36 (10.5%)	30 (10.7%)	10 (14.9%)	0.559
Total number of CLN	8.83 ± 5.75	9.16 ± 6.14	8.67 ± 4.61	0.715
Number of metastatic CLN	1.70 ± 2.41	1.64 ± 2.37	1.84 ± 2.57	0.827
WBC (×10^9^/L)	9.2 ± 2.7	9.5 ± 4.7	9.9 ± 2.9	0.330
Postoperative CRP (mg/L)	8.3 ± 7.0	9.3 ± 8.3	9.4 ± 7.7	0.172
Operative time (min)	116.2 ± 44.3	118.5 ± 46.6	121.7 ± 40.3	0.601
Mandibular swell	4 (1.2%)	2 (0.7%)	7 (10.4%)	<0.01
Visual VAS	1.7 ± 0.5	1.7 ± 0.5	2.1 ± 0.6	<0.01
Hospital stay (days)	3.89 ± 1.09	3.99 ± 1.07	3.88 ± 1.12	0.515
Quality of life	7.89 ± 0.80	7.94 ± 0.71	7.88 ± 0.69	0.609

EMG, electromyography; PTH, parathyroid hormone; CLN, central lymph nodes; WBC, white blood cell; CRP, C-reactive protein; VAS, visual analogue scale.

### Surgical Complications

The surgical complications, including postoperative vocal cord paralysis and hypocalcemia were recorded. The electromyography (EMG) changes during the surgery, and the complains of hoarseness and laryngoscope examinations postoperatively were recorded. As shown in **Table 2**, there was no significant difference in the comparison of EMG changes (8.2% *vs*. 7.5% *vs*. 3.0%, *P* = 0.330) and post-operative transient vocal cord paralysis (2.0% *vs*. 3.2% *vs*. 1.5%, *P* = 0.563) in the groups of A, B, and C type. No permanent vocal cord paralysis occurred in all the three groups. The levels of PTH and serum calcium were recorded at the next day postoperatively. It was similar in PTH levels (34.7 ± 16.5 *vs*. 36.0 ± 18.4 *vs*. 35.7 ± 18.9, *P* = 0.642) and serum calcium (2.09 ± 0.14 *vs*. 2.11 ± 0.13 *vs*. 2.08 ± 0.13, *P* = 0.360) in the comparison of type A, B, and C. The incidences of numbness in limbs were also similar in the comparison of type A, B, and C (10.5% *vs*. 10.7% *vs*. 14.9%, *P* = 0.559).

### Comparison of Lymph Node Dissection in the Central Compartment

The total number and metastatic central lymph nodes (CLN) were recorded. It was similar in the total number of CLN in the comparison of group A, B, and C (8.83 ± 5.75 *vs*. 9.16 ± 6.14 *vs*. 8.67 ± 4.61, *P* = 0.715). And no difference was found in the number of metastatic CLN in the comparison of group A, B, and C (1.70 ± 2.41 *vs*. 1.64 ± 2.37 *vs*. 1.84 ± 2.57, *P* = 0.827).

### Comparison of Postoperative Inflammatory Response

WBC and CRP were recorded to assess the postoperative inflammatory response. There was no difference in the number of WBC in the comparison of group A, B, and C (9.2 ± 2.7 *vs*. 9.5 ± 4.7 *vs*. 9.9 ± 2.9, *P* = 0.330). And it was similar in postoperative CRP level in the comparison of group A, B, and C (8.3 ± 7.0 *vs*. 9.3 ± 8.3 *vs*. 9.4 ± 7.7, *P* = 0.172).

### Operative Assessment and Qof Evaluation in the Follow-Up

The operative time and postoperative hospital stay were recorded. And the Quality of life (Qof) was assessed at 3 months postoperatively during the follow-up. The operative time was similar in the three groups of type A, B, and C (116.2 ± 44.3 *vs*. 118.5 ± 46.6 *vs*. 121.7 ± 40.3, *P* = 0.601). And it was similar in the comparation of postoperative hospital stay in the three groups (3.89 ± 1.09 *vs*. 3.99 ± 1.07 *vs*. 3.88 ± 1.12, *P* = 0.515). There was no difference in Qof assessment in the three groups of type A, B, and C (7.89 ± 0.80 *vs*. 7.94 ± 0.71 *vs*. 7.88 ± 0.69, *P* = 0.609).

## Discussion

Since a large case series of transoral endoscopic thyroidectomy was reported in 2016, multiple hospital centers worldwide have introduced this technique and reported their initial experience (7). Up to now, transoral endoscopic thyroidectomy *via* vestibular approach (TOETVA) has become one of the most widely used techniques in scarless endoscopic thyroidectomy (8). Our group has reported a large cohort of TOETVA cases, in which we demonstrated that transoral endoscopic thyroidectomy has the same surgical safety compared with total endoscopic thyroidectomy *via* areola approach, and conventional open thyroidectomy (9). Recently, it has been found by us that the surgical outcomes of transoral endoscopic thyroidectomy are safe in the treatment of PTC with a diameter between > 1cm and ≤ 3.5cm (3).

Hence, we compared the different classified types of mandibular jawlines on TOETVA. It was found safe of TOETVA in the treatment of PTC patients with all the three types of mandibular jawlines. No case was needed to transverse conventional open choice in all the 690 cases. No significant difference was found in the comparison of surgical complications in the types of A, B, and C, including postoperative vocal cord paralysis and hypoparathyroidism. Mental nerve injury is a unique complication of TOETVA (10). And the incidence of mental nerve injury is quite diverse (11). In our institute, only 2 cases of mental nerve injury were detected at the initial period after TOETVA performed. There was no mental nerve injury found in this study of 690 PTC cases. These results might provide evidence mental nerve injury rarely occurs with TOETVA in all the patients of class A, B, and C mandibular jawline.

The effect and feasibility were also verified with the largest case series of TOETVA for PTC in the present study. Total and metastatic number of central lymph nodes were similar in the comparison of all the three types, which represents the effect was similar during the implementation of TOETVA in the patients of type A, B, and C. The postoperative inflammatory response, including WBC and CRP, were recorded similar, which represents the feasibility of TOETVA was comparable in type A, B and C.

According to the results of comparative analyses, the length of jaw in the patients of type C was significantly more than type A and B. Due the longer jawline, it had more difficulty in maintaining the surgical space, and the ratios of using suspension assisted system in the patients of type C were also higher than type A and B significantly. Additionally, patients of type C experienced a higher ratio of mandibular swell, and the mandibular swell would be absorbed at 7 ~ 10 days post TOETVA. Patients of type C had higher scores of VAS at the next day post TOETVA, and the pain feelings would be reduced at 3 ~ 5 days post TOETVA according to our experience. Moreover, there was no difference found in the comparison of Qof in the period of follow-up, which represents the longtime postoperative feelings were similar in the comparison of type A, B, and C. Furthermore, several innovative techniques were implemented in TOETVA, which was useful to maintain the surgical space, especially in the patients of type C.

### Injecting Epinephrine Solution

Epinephrine solution of diluted adrenaline solution (1:500 000) was injected from the vestibule to the anterior of the neck at the subcutaneous layer (12). The uneven surface of the mandibular would be topped up by the epinephrine solution. Additionally, diluted adrenaline contributed to microvascular constriction, which would be helpful to reduce bleeding during the process of space creating.

### Real-Time Observation

We firstly designed a visual separation device, which was used for the separation of skin flap (13). The visual separation bar has a see-through head, could be used for real-time observation during blunt separation. The correct fascia layer is shown white, while a yellow appearance means over superficial or fat layer, and red appearance been over deep or muscle layer. With this help, the tissue separation layer could be adjusted at any time during the process of flap dissection. Additionally, anterior jugular microvascular could also be observed, which would be helpful to avoid bleeding in the procedure of building working space.

### Hybrid Space-Maintaining Method

The hybrid space-maintaining system combining constant low pressure gas insufflation with a flap lifting device. We have reported this method before, which was described briefly in the following (14). During the operation, the CO_2_ was insufflated and maintained at a low pressure of 6 mm Hg. Additionally, the flap was lifted to expand the space by wires or silk sutures, which retracted by a suspension system. As the critical device of suspension system, a disinfected L-shaped pole was wrapped by a sterile protective cover and then fixed on the side of the head as the suspension frame.

### Add Another Vacuum Tube for Working Space Building

**Figure 3:**
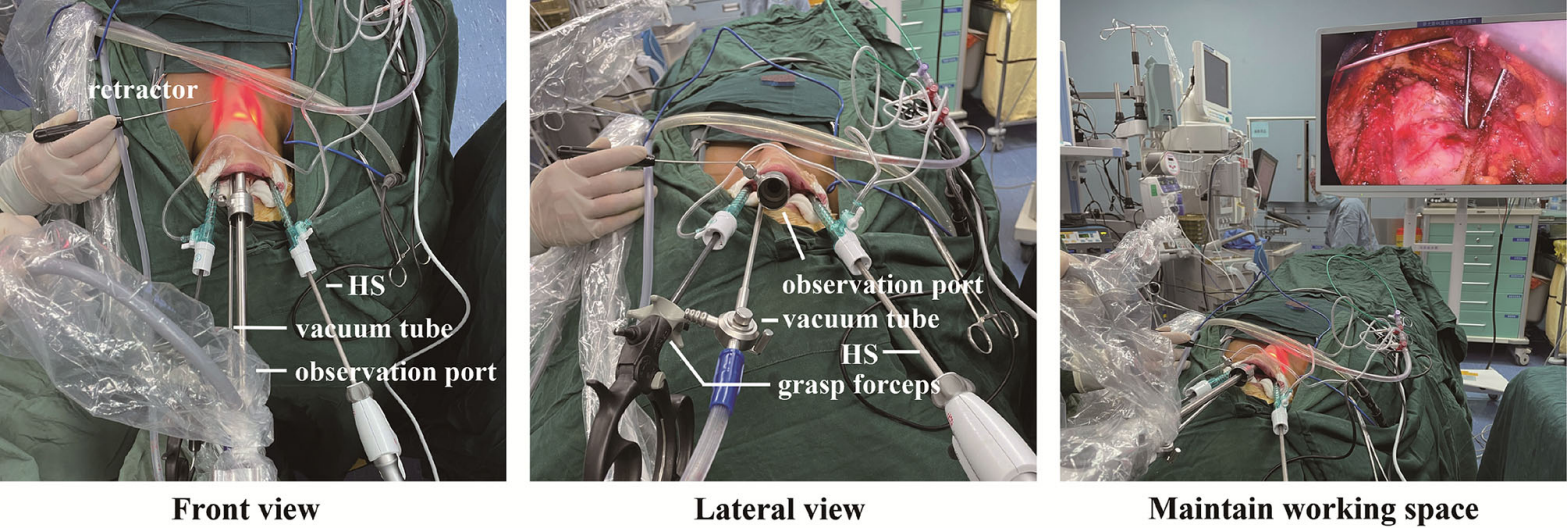
Introduce another vacuum tube for working space building in TOETVA.

The middle curvilinear incision was expanded to 2 cm, which used for observation port and another vacuum tube. Vacuum tube shore up and air inhalation were alternatively used for flap suspension during working space building. Order to reduce the smoke circumstance, the operation space was kept a negative pressure during the whole process. Additional vacuum tube was placed next to the observation port for flap suspension to keep the operation space under negative pressure **(** **Figure 3** **)**. And the vacuum tube could also be used for air inhalation when much smoke generated in the surgical circumstance.

### The Advantages and Disadvantages of TOETVA

**Figure 4:**
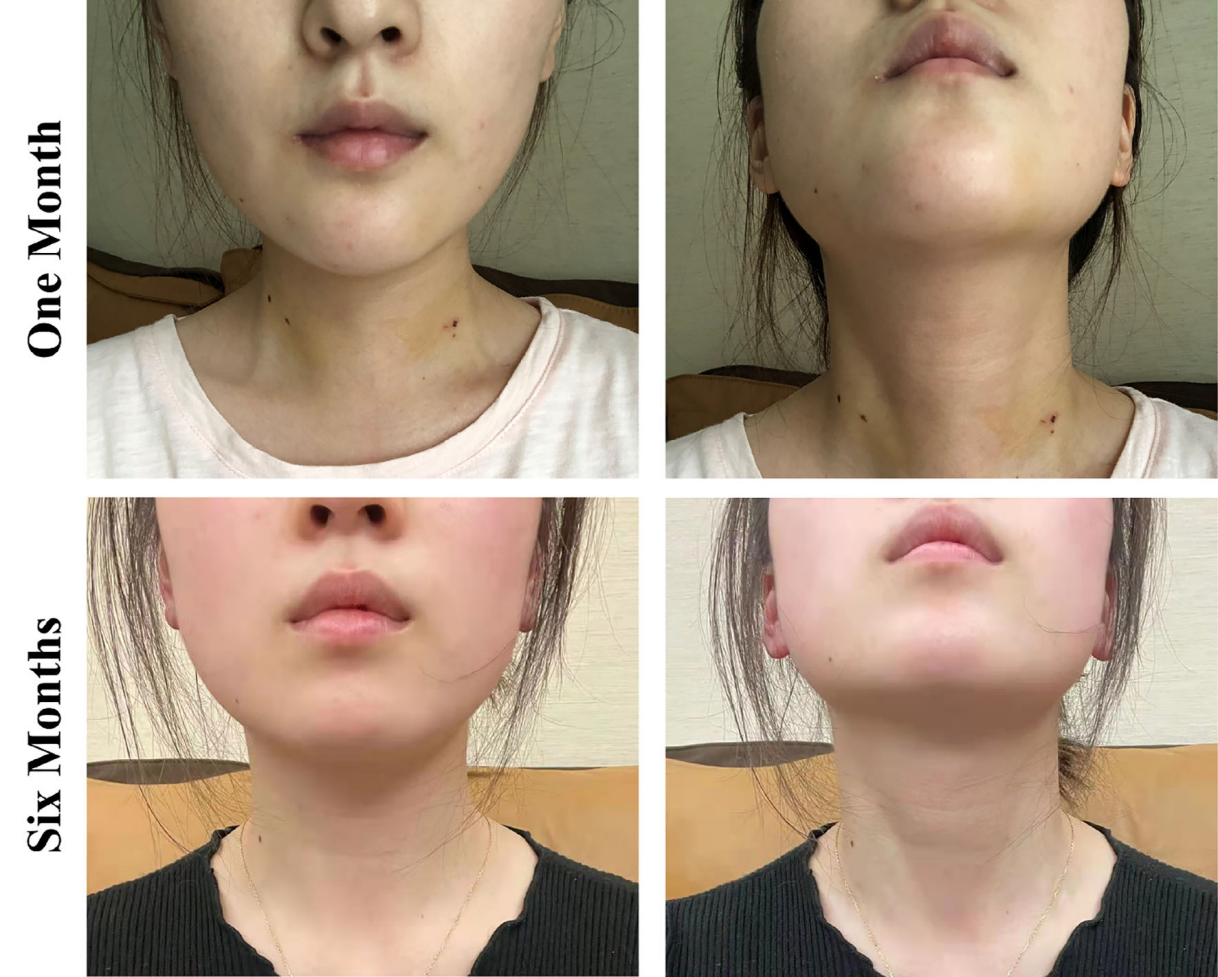
Cosmetic results 1 month and 6 months postoperatively of TOETVA.

TOETVA provides a cranial-caudal perspective that can better expose and more completely central nodes dissection, especially for the lower part including levels VI and VII (15). However, it increases difficulty of handling the thyroid superior pole at the beginning, identifying the superior thyroid artery and superior laryngeal nerve (SLN) (16). Due to the limitations, it is important to evaluate whether different mandibular jawline types would increase the surgical complications in TOETVA. In this study, all tumors were completely removed, and no skin flap disruption or permanent SLN injury occurred. And the different mandibular jawline types, especially for type C, can be overcome with the development of operative proficiency and the improvement of surgical instruments. Additionally, patients chosen TOETVA had much more scores of Qof (17), especially in the aspect of cosmetic result (18), which can hide incisions in the mouth without any scars on the body appearance **(** **Figure 4** **)**.

Our study has some limitations. Firstly, it was a retrospective study, we only enrolled patients who had a pathological diagnosis of PTC from January 2015 to June 2020 at our single center. Secondly, the operation choices of ipsilateral lobectomy or total thyroidectomy, and central node dissections were according to Chinese guidelines. Additionally, all cases were performed within 18 – 36 months period, and the follow-up time was not long enough to observe tumor recurrence.

## Conclusion

A new classification was introduced in TOETVA, so as to evaluate the influence of different mandibular jawlines. It has been found that the safety, effect and feasibility were similar in the treatment of PTC with TOETVA in all the mandibular jawlines of type A, B, and C. However, patients of type C would experience a higher ratio of mandibular swell, and higher scores of VAS post TOETVA. New techniques, including real-time observation, hybrid space-maintaining, and adding another vacuum tube may be helpful for working space building of TOETVA, especially in the patients of type C.

## Data Availability Statement

The original contributions presented in the study are included in the article/supplementary material. Further inquiries can be directed to the corresponding authors.

## Ethics Statement

Written informed consent was obtained from the individual(s) for the publication of any potentially identifiable images or data included in this article.

## Author Contributions

XY, YJ, and YW substantial contributed to conception and design. YL, QH, YJ, LP, and PZ contributed to acquisition of data. YL analysis and interpretation of data. YW and XY contributed to draft the article. All authors contributed to the article and approved the submitted version.

## Funding

This study is financially supported by Health Innovation Talents Project of Zhejiang Province (2021RC004) and Basic Public Welfare Research Project of Zhejiang Province (LGF22H070002).

## Conflict of Interest

The authors declare that the research was conducted in the absence of any commercial or financial relationships that could be construed as a potential conflict of interest.

## Publisher’s Note

All claims expressed in this article are solely those of the authors and do not necessarily represent those of their affiliated organizations, or those of the publisher, the editors and the reviewers. Any product that may be evaluated in this article, or claim that may be made by its manufacturer, is not guaranteed or endorsed by the publisher.
